# Supplementary material for: Causal Relationship between Aging and Anorexia Nervosa: A White-Matter-Microstructure-Mediated Mendelian Randomization Analysis
Source: Biomedicines. 2024 Aug 16;12(8):1874. doi: 10.3390/biomedicines12081874 (PMC11351342; doi:10.3390/biomedicines12081874)
Supplement: Supplementary file 1 [file biomedicines-12-01874-s001.zip › Supplementary Figure/Figure S8/Leaveoneout plot of MR analysis for TL to ubm-b-1520.pdf]

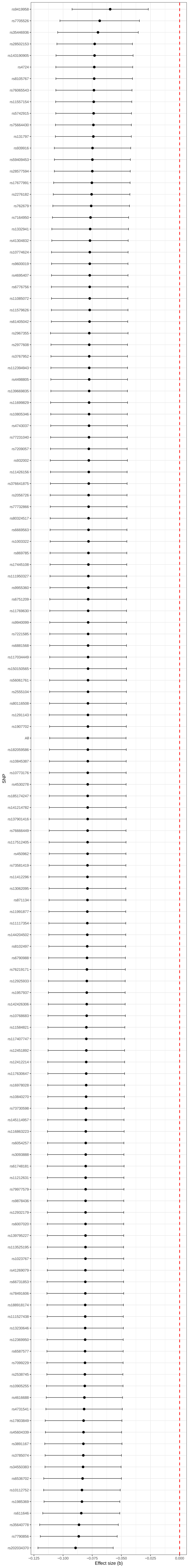
rs9419958
rs7705526
rs35446936
rs28502153
rs143190905
rs4724
rs8105767
rs76065543
rs11557154
rs5742915
rs75664430
rs131797
rs939916
rs59409453
rs28577594
rs17677991
rs2276182
rs762679
rs7164950
rs1332941
rs41304832
rs10774624
rs9600019
rs4695407
rs6776756
rs11085072
rs11579626
rs61405042
rs2967355
rs2977608
rs3767952
rs112394943
rs4498805
rs139669835
rs11699829
rs10805346
rs4743037
rs77231040
rs7209057
rs932002
rs11426156
rs376641875
rs2056726
rs77732866
rs80324517
rs6669563
rs1003322
rs869785
rs17445108
rs111950327
rs9955360
rs6751209
rs11769630
rs9940099
rs7221585
rs6881568
rs117034449
rs150150565
rs56061761
rs2555104
rs80116508
rs1291143
rs1907702
All
rs182059586
rs10845387
rs10773176
rs4530278
rs185174247
rs141214782
rs137901416
rs76066449
rs117512405
rs450962
rs73581419
rs11412296
rs13062095
rs871134
rs11991877
rs11117354
rs144204502
rs8102497
rs6790988
rs76219171
rs12925933
rs1957937
rs142426306
rs10768683
rs11584821
rs117407747
rs12451892
rs12412214
rs117630647
rs16978028
rs10840270
rs73730598
rs145114957
rs116863223
rs6054257
rs3093888
rs61748181
rs11212631
rs79077579
rs9878436
rs12932179
rs6007020
rs139795227
rs113525195
rs1023767
rs41269079
rs66731853
rs78491606
rs188918174
rs111527438
rs13230646
rs12369950
rs6587577
rs7099229
rs2538745
rs10905255
rs4616688
rs4731541
rs17803849
rs45604339
rs3891167
rs3785074
rs34550383
rs6536702
rs10112752
rs1985369
rs611646
rs35640778
rs7790856
rs202034370
SNP
-0.125
-0.100
-0.075
-0.050
-0.025
0.000
Effect size (b)
